# Enhancing the Thermal Resistance of UV-Curable Resin Using (3-Thiopropyl)polysilsesquioxane

**DOI:** 10.3390/ma17102219

**Published:** 2024-05-08

**Authors:** Daria Pakuła, Bogna Sztorch, Monika Topa-Skwarczyńska, Karolina Gałuszka, Joanna Ortyl, Bogdan Marciniec, Robert E. Przekop

**Affiliations:** 1 Faculty of Chemistry, Adam Mickiewicz University in Poznań, ul. Uniwersytetu Poznańskiego 8, 61-614 Poznan, Poland; darpak@amu.edu.pl; 2 Centre for Advanced Technologies, Adam Mickiewicz University Poznan, ul. Uniwersytetu Poznanskiego 10, 61-614 Poznan, Poland; bogna.sztorch@amu.edu.pl; 3 Faculty of Chemical Engineering and Technology, Cracow University of Technology, ul. Warszawska 24, 31-155 Krakow, Poland; monika.topa-skwarczynska@pk.edu.pl (M.T.-S.); karolina.galuszka26@gmail.com (K.G.); joanna.ortyl@pk.edu.pl (J.O.)

**Keywords:** (3-thiopropyl)polysilsesquioxane, urethane-acrylate resin, composites, thermal properties

## Abstract

This study delineates a methodology for the preparation of new composites based on a photocurable urethane-acrylate resin, which has been modified with (3-thiopropyl)polysilsesquioxane (SSQ-SH). The organosilicon compound combines fully enclosed cage structures and incompletely condensed silanols (a mixture of random structures) obtained through the hydrolytic condensation of (3-mercaptopropyl)trimethoxysilane. This process involves a thiol-ene “click” reaction between SSQ-SH and a commercially available resin (Ebecryl 1271^®^) in the presence of the photoinitiator DMPA, resulting in composites with significantly changed thermal properties. Various tests were conducted, including thermogravimetric analysis (TGA), Fourier transmittance infrared spectroscopy (FT-IR), differential scanning calorimetry (Photo-DSC), and photoreological measurement mechanical property, and water contact angle (WCA) tests. The modification of resin with SSQ-SH increased the temperature of 1% and 5% mass loss compared to the reference (for 50 wt% SSQ-SH, T_5%_ was 310.8 °C, an increase of 20.4 °C). A composition containing 50 wt% of SSQ-SH crosslinked faster than the reference resin, a phenomenon confirmed by photorheological tests. This research highlights the potential of new composite materials in coating applications across diverse industries. The modification of resin with SSQ-SH not only enhances thermal properties but also introduces a host of functional improvements, thereby elevating the performance of the resulting coatings.

## 1. Introduction

**Figure 1:**
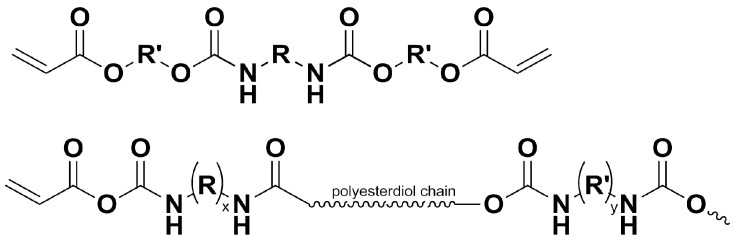
Example structures of urethane-acrylic resin oligomers.

The utilization of photocurable resins has garnered interest in both scientific and industrial domains [1]. Due to the wide range and availability of substrates, fast curing times, low emission of volatile organic compounds generated during the process, low energy consumption, and ease of reaction, these resins are used in many industries [2,3]. Since the first photo-curable coating was patented, these materials have been the subject of extensive research [4]. UV-curable materials are used in various applications, including as protective coatings for metal surfaces [5] and wood [6,7], adhesives [8,9], inks [10,11], and biomaterials [3,12,13,14], as well as in dentistry [15,16,17] and the automotive industry [18,19,20]. One of the primary resins utilized in UV-curable materials is urethane-acrylate resin (Figure 1). The final product’s properties depend on the type of substrate used, functional groups, curing time, and the choice of reaction conditions, such as temperature and the solvent or initiator type and amount [21,22,23,24]. Aliphatic urethane-acrylate coatings exhibit higher flexibility and weather resistance compared to aromatic polymers [25,26]. It is relatively easy to alter these systems’ properties, such as by shortening the processing time and lowering production costs or improving performance parameters, leading to innovative solutions and new applications.

Lately, there has been growing interest in organosilicon compounds due to their ability to modify various properties of plastics [27,28]. Silsesquioxanes are hybrid inorganic–organic compounds [29,30]. They can create various spatial structures, among which the most popular are cage T_8_ units consisting of a stable cubic core composed of alternately arranged Si-O-Si units and reactive or inert functional groups attached to the corners of the cage [31,32]. Adding organosilicon compounds to a polymer matrix can change rheological and hydrophobic properties [33], thermal stability [34], mechanical strength [35], and chemical resistance [36]. In the scientific literature, there are examples of the modification of photocurable urethane-acrylate resins with silsesquioxanes [37]. The application of octa(methacryloxypropyl)silsesquioxane (M-POSS) in an amount of 5–35% by weight to commercial urethane-acrylate resin significantly changed the properties of the composites, including in the form of an increase in hardness by about 56%, an improvement in tensile strength, and an increase in thermal stability [38]. Acrylic paint coatings have been modified with silsesquioxanes containing reactive groups (methacrylic groups) and non-reactive fluoroalkyl groups in order to bestow increased scratch resistance and higher hydrophobicity. Higher hydrophobicity is caused by the presence of derivatives with fluoroalkyl groups on the surface (slip layer) [39]. (3-thiopropyl)polysilsesquioxane contains reactive -SH groups in its structure that can easily participate in a click reaction, forming a 3D polymer network. Thiol compounds have been employed as precursors of organic–inorganic nanocomposites. A. Lungu et al. synthesized thiol-epoxy materials using a commercial epoxy resin with a trifunctional (3SH) or tetrafunctional (4SH) thiol as a cross-linking agent (epoxy:thiol ratio = 1:1). POSS-(3-glycidylpropoxy)heptaisobutyl (Ep-POSS) or S-(3-mercaptopropyl)heptaisobutyl silsesquioxane (SH-POSS) were used to replace 10% of the corresponding chemical functional equivalent in the thiol-epoxy [40]. The addition of silsesquioxane with thiol groups generated a plasticizing effect, which led to a decrease in the degree of cross-linking and, as a result, an increase in the flexibility of the composite [40]. Octa(mercaptopropyl)polyhedral oligomeric silsesquioxane in epoxy nanocomposites (diglycidyl ether of bisphenol A—DGEBA) influenced the impact properties of epoxy resins (based on an impact test). The highest impact strength value (an increase of 5 kJ/m^2^ relative to the base resin) was recorded for a concentration of 20 wt% 8SH-POSS. MPOSS/DGEBA nanocomposites are characterized by better thermal stability at high temperatures [41]. E. Çakmakçı reported the effect of allylamino diphenylphosphine oxide (AADPPO or allyl phosphinic amide-APA) incorporating 8SH-POSS on the flame resistance of epoxy-acrylate resin. 8SH-POSS significantly increased the conversion of double bonds in the system [42]. The presented examples indicate the wide range of applications of compounds with thiol groups, which can react with, e.g., epoxy or (meth)acrylic groups, leading to the formation of new composites and significantly improved properties. However, no information was found in the available scientific literature regarding the modification of urethane-acrylic resins using (3-thiopropyl)polysilsesquioxanes containing SH groups in their structures, prompting research in this area. The addition of organosilicon compounds containing thiol groups allows for the formation of bonds between acrylic groups and SH through click reactions, contributing to changes in physicochemical properties, including thermal stability.

This paper presents the procedure for obtaining photocurable aliphatic urethane-acrylate resins modified with (3-thiopropyl)polysilsesquioxane, which is the product of the hydrolytic condensation of (3-mercaptopropyl)trimethoxysilane. SSQ-SH is a mixture of fully closed cage structures and incompletely condensed silanols. By means of a thiol-ene click reaction between SSQ-SH and a commercially available resin (Ebecryl 1271^®^) in the presence of the photoinitiator DMPA, composites with significantly changed physicochemical properties were obtained. To evaluate the properties of these composites, various tests, such as thermogravimetric analysis (TGA), differential scanning calorimetry (Photo-DSC), and photoreological measurement, Fourier infrared spectroscopy (FT-IR), mechanical property, and water contact angle (WCA) tests, were performed.

## 2. Materials and Methods

### 2.1. Chemicals

The chemicals used and the companies from which they were purchased are as follows: 3-mercaptopropyltrimethoxysilane (99%) and methanol p.a. were obtained from P.P.H. Stanlab; hydrochloric acid (35–38%), toluene, tetrahydrofuran, and dichloromethane were obtained from Chempur; 2,2-dimethoxy-2-phenylacetophenone (DMPA) and chloroform-d were obtained from Merck Group (Darmstadt, Germany); and difunctional aliphatic urethane acrylate resin (Ebecryl 1271^®^) was obtained from Allnex (Frankfurt am Main, Germany).

### 2.2. Analytical Methods and Methodology

Fourier-transform infrared spectroscopy (FT-IR) spectra were collected at ambient temperature using a Nicolet iS50 FT-IR Spectrometer (Thermo Fisher Scientific, Waltham, MA, USA) equipped with a diamond ATR unit with a resolution of 4 cm^−1^. The spectral range was from 4000 to 400 cm^−1^, and the spectra represent an average of 16 scans. FT-IR measurements of residues after thermal analysis were carried out in transmittance mode. The standard KBr pellet method was used. A total of 32 scans in the range of 4000–400 cm^−1^ were collected, with 2 cm^−1^ resolution.

Thermogravimetric analysis (TGA) was conducting using a NETZSCH 209 F1 Libra thermogravimetric analyzer (Selb, Germany). Samples weighing 4.0 ± 0.2 mg for thermal analysis and 15.0 ± 0.2 mg for microscopic analysis were placed in Al_2_O_3_ crucibles. The experiments were carried out in a nitrogen atmosphere (flow rate of 20 mL/min) within a temperature range of 30–950 °C and with a 10 °C/min heating rate.

Images of the residue following the thermal decomposition of resin were taken using Keyence VHX-7000 digital microscope (Keyence International, Belgium, NV/SA, Mechelen, Belgium) with VH-Z100R wide-angle zoom lens at 100× magnification. The images were acquired utilizing depth composition techniques assisted by 3D imaging software (System ver 1.05) integrated into the microscope system.

Water Contact Angle (WCA) analyses were conducted using the sessile drop technique under ambient conditions with respect to room temperature and atmospheric pressure using a Krüss DSA100 goniometer. Each sample underwent three separate measurements, each involving a 5 μL water droplet, and the obtained results were averaged to reduce the impact of surface non-uniformity.

Photoreological measurements of the developed compositions were carried out using a Physica MCR-302 rheometer (Anton Paar, Torrance, CA, USA). A UV-LED 365 nm Bluepoint LED eco (Hönle UV Technology, Gräfelfing, Germany) was applied for irradiation. The diode’s intensity was consistently tuned at 15 mW·cm^−2^. The two plates in the experiment were spaced 0.5 mm apart, and the frequency and amplitude were maintained at 10 Hz and 1%, respectively. To steady the apparatus, the light was turned on ten seconds after the measurement began. 

Differential scanning calorimetry (Photo-DSC) measurements were carried out using a Photo-DSC 204 F1 Phoenix (Netzsch-Gerätebau GmbH, Selb, Germany). At this juncture, 2.0 ± 0.5 mg samples of each composition were placed in aluminum crucibles. The UV-LED 365 nm Bluepoint LED eco (Hönle UV Technology, Germany, the same device used for the photorheological studies) was used as the light source. The light intensity at the end of the fiber was measured with an OmniCure R2000 radiometer (Excelitas Technologies, Mississauga, ON, Canada), and this value was 15 mW·cm^−2^. All measurements were carried out in an inert atmosphere (nitrogen flow rate was 20 mL·min^−1^) and under isothermal conditions at 25 °C. The amount of heat released during the polymerization process was recorded as a function of time.

Tensile strength tests were conducted using an Instron 5969 Universal Testing Machine (Instron, Darmstadt, Germany), following the guidelines outlined in the EN ISO 527-2:1996 standard [43]. The traversal speed was set to 50 mm/min.

### 2.3. Synthesis of Precursor Octa(3-thiopropyl)silsesquioxane

**Figure 2:**
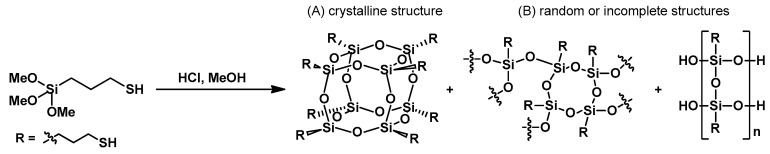
Hydrolytic condensation of (3-thiopropyl)trimethoxysilane: (**A**) crystalline product; (**B**) mixture of different structures.

The synthesis of (3-thiopropyl)polysilsesquioxane was conducted following the procedures outlined in the literature [44,45]. A viscous oil (hereinafter referred to as SSQ-SH) was employed for resin modification. SSQ-SH is composed of various structures resulting from random or incomplete condensation processes, along with suspended fine crystallites (Figure 2). For SSQ-SH, the signals at −56.37–(−60.38) (Si–OH) and −64.42–(−67.15) (Si–O–Si) observed in the ^29^Si NMR indicate a mixture of different structures constituting the products of random or incomplete condensation. Details regarding the spectroscopic analysis of SSQ-SH can be found in our previous publication [46].

### 2.4. General Procedure for Composite Preparation

The thiol-ene reactions between resin and SSQ-SH were performed under an air atmosphere using 5 mL vials equipped with magnetic stirrers. SSQ-SH, resin, 3 mL of toluene, and 1 wt% DMPA relative to the weight of resin/SSQ-SH were placed in the vials. The mixture was stirred vigorously for 5 min. Following this, the samples underwent degassing in a vacuum chamber to eliminate air bubbles. Subsequently, the mixture was poured into a Petri dish and exposed to UV radiation at a wavelength of λ = 365 nm for one hour. All data for obtained coatings with different SSQ-SH concentrations are presented in Table 1. Composite materials were obtained as described in the patent application [47]. To prepare samples for mechanical testing, analogous compositions were recalculated for a 50 g system. The preparation of normalized specimens with a size of 5A was performed by cutting them using a sample cutter (Instron, Norwood, MA, USA) with manual drive.

## 3. Results and Discussion

### 3.1. FT-IR-ATR Spectroscopy Analysis

**Figure 3:**
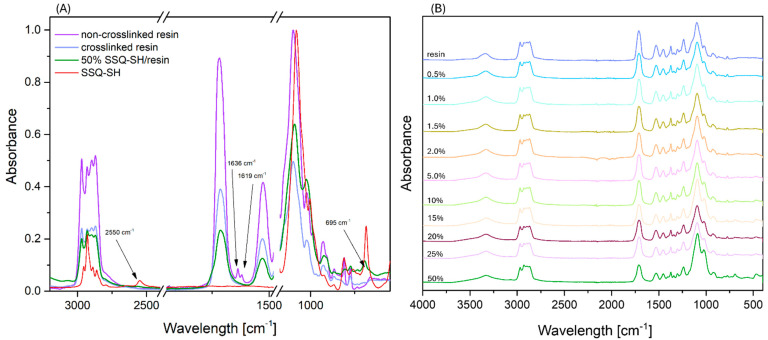
FT-IR spectra of resin and composites; (**A**) selected spectra; (**B**) all composites.

Figure 3A displays the FT-IR spectra of the non-crosslinked and crosslinked resin, as well as spectrum of the resin modified with SSQ-SH at a 50% weight concentration, and the spectrum of the organosilicon modifier. The data for the non-crosslinked resin indicate the presence of two characteristic absorption bands at 1636 and 1619 cm^−1^, which can be attributed to acrylic C=C bonds [48,49]. These bands were utilized as reference points during the experiments, serving as indicators of reaction progress during the reaction. Both for the crosslinked unmodified resin and the SSQ-SH/resin composites, the complete disappearance of these bands was observed, confirming the conversion of the substrates. In the spectrum of the organosilicon modifier, a characteristic band at around 2550 cm^−1^, ascribed to the -SH group, was evident. However, this band was not observed in the spectra of the modified composites, further confirming that a “click” reaction occurred between the thiol and double bond in the resin. Figure 3B displays the FT-IR-ATR spectra of all the obtained cross-linked SSQ-SH/resin composites. Each composite spectrum shows bands that are characteristic of the resin matrix (Ebecryl^®^). The bands characteristic of urethane groups in the range of 3300–3450 cm^−1^ confirm the presence of N-H groups (stretching vibrations ascribed to secondary amides), and the stretching band at 1713 cm^−1^ confirms the presence of C=O groups. As the modifier concentration in the composites increases, the intensity of bands at 1713 cm^−1^ decreases, which is linked to the increasing percentage of the SSQ-SH modifier. New bands originating from SSQ-SH can be observed in the spectra of the modified systems. The spectra exhibit bands originating from silicon–oxygen vibrations, including the deformational stretching at 468 cm^−1^ (δ(OSiO)), symmetric stretching in the range of 780–550 cm^−1^ (ν*_s_*(SiOSi)), and antisymmetric stretching within the 1100–1000 cm^−1^ range (ν*_as_*(SiOSi)). Notably, the intensity of these bands escalates with the augmentation in the quantity of the modifier. In addition, a band at 774 cm^−1^ is visible, confirming the presence of vibrations occurring between Si and C. The band observed at 695 cm^−1^ originates from the C-S bond present in the silsesquioxane structure [50]. Moreover, new C-S bonds form between the resin and the thiol during the reaction, which complicates the unequivocal interpretation of reaction occurrence based on this band. For all modified and reference samples, bands from C-H stretching vibrations in the 2990–2850 cm^−1^ range were observed for the modifier and the resin [51]. 

### 3.2. Thermogravimetric Analysis

**Figure 4:**
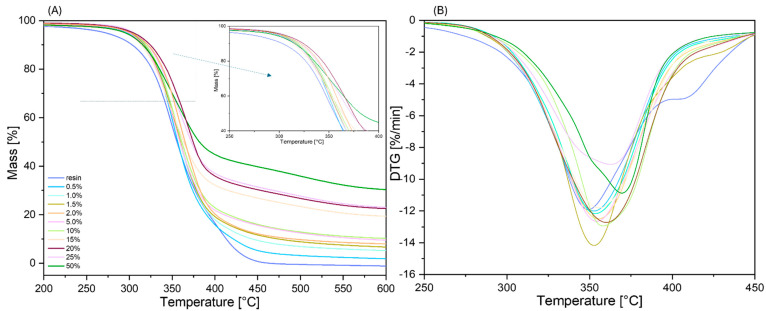
(**A**) TGA curves recorded for samples under nitrogen flow; (**B**) DTG curves of measured samples.

**Figure 5:**
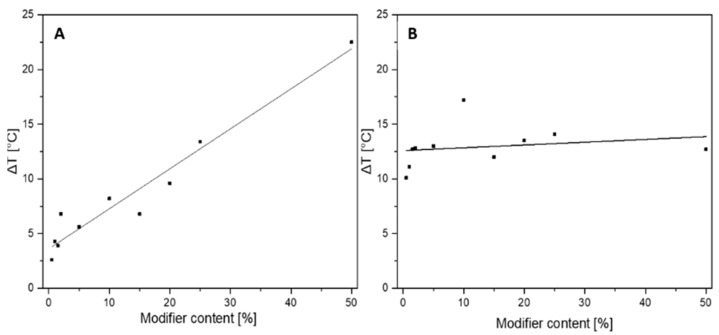
Δ*T* for (**A**) temperature at the maximum rate of mass loss [°C]; (**B**) onset temperature [°C].

To assess the thermal stability of the composites and compare them to the unmodified resin, TGA analysis in a nitrogen atmosphere was conducted (Figure 4). Based on the results obtained from TGA and DTG, the temperature of 1% mass loss, the temperature of 5% mass loss, the onset temperature, and the temperature at the maximum rate of mass loss and residual mass (Table 2) were determined. Δ*T* was calculated based on the difference between the temperatures for the modified samples and the reference sample. Figure 5 illustrates the *ΔT* values along with a linear fit. This analysis revealed that the addition of SSQ-SH to the resin caused a significant shift in the thermal decomposition temperature towards higher values. The beginning of thermal decomposition, i.e., the “onset”, for the modified samples was also observed at higher temperatures. For the system with 50 wt% SSQ-SH, the onset temperature was 12.7 °C higher than that for the unmodified system. The initial mass loss occurred at higher temperatures in relation to the base system and also for samples with a low content of the organosilicon modifier, i.e., 0.5 wt% SSQ-SH. A similar effect was also observed for the “onset” temperature (an increase of 11.1 °C over the reference). Additionally, modifying the resin with SSQ-SH affected the temperature change for both 1% and 5% mass loss compared to the reference. For instance, the temperature at 5% mass loss for 50 wt% SSQ-SH was 310.8 °C, indicating a 20.4 °C increase compared to that for unmodified Ebecryl^®^ resin. The click reaction between the acrylic resin and the -SH thiol group facilitated the formation of new bonds, resulting in a 3D polymer network that strengthened the material’s structure, thereby improving its thermal stability. The high stability of the organosilicon precursor also contributed to the increase in T_1%_, T_5%_, and onset temperatures. The onset temperature for the neat organosilicon compound SSQ-SH is 340 °C. The thermal decomposition of SSQ-SH was presented in our previous work [46]. With the increase in SSQ-SH concentration, an increase in residual masses became apparent. The residual mass at 600 °C for the 50 wt% SSQ-SH/resin was 30.3%, indicating the presence of residual silica and coke.

#### 3.2.1. Microscopic Analysis

**Figure 6:**
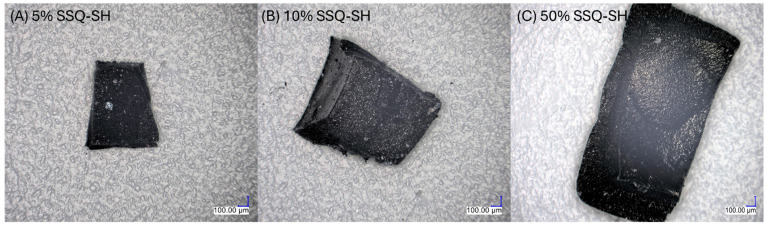
Microscopic images of residual mass.

A microscopic analysis was conducted on the residual samples subsequent to conducting TGA thermogravimetric tests. Figure 6 depicts pictures of residues for selected concentrations of 5%, 10%, and 50% of the modifier. The remaining mass comprises silica and coke. There is potential for the formation of carbides (SiC), distinguished by a vitreous structure of the material, particularly evident with the 50% weight modification regarding SSQ-SH. FT-IR analysis was conducted in transmittance mode to confirm the composition of the residue.

#### 3.2.2. FT-IR Spectroscopy Analysis after Thermal Decomposition

**Figure 7:**
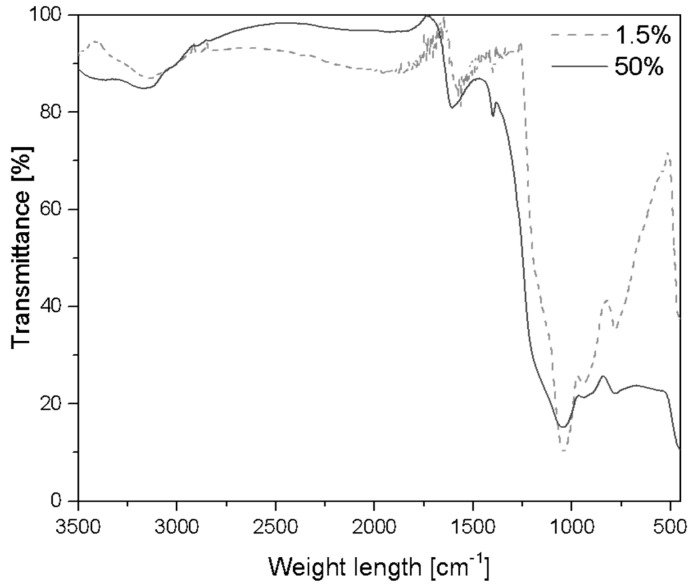
FT-IR spectra after thermal decomposition TGA.

An FT-IR spectroscopy analysis on the residual mass of selected concentrations of SSQ-SH in the resin (1.5 wt% and 50 wt%) was conducted. A total of 1.5 mg of the residue was mixed with 200 mg of KBr in a mortar (via grinding), and the FT-IR spectrum was recorded in transmittance mode. As shown in Figure 7, the residual mass spectra after thermal analysis revealed bands at 1100–1030 cm^−1^, indicating the presence of asymmetric SiOSi stretching vibrations. Additionally, out-of-plane deformation vibration of SiOSi were assigned for the range of 450–420 cm^−1^. The bands at 778 cm^−1^ were attributed to stretching vibrations of the Si-C bonds, which may indicate the process of carbonization [52]. 

### 3.3. Photoreological and Kinetic Studies of the New Compositions

**Figure 8:**
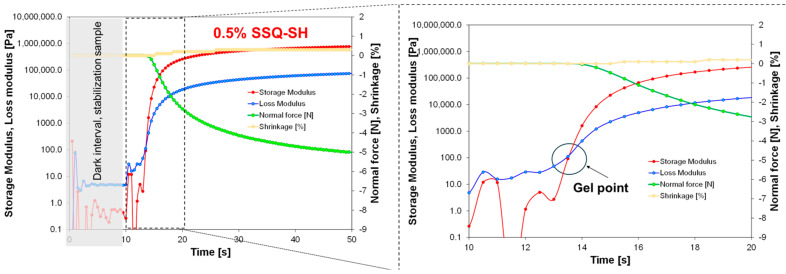
Storage modulus (red), loss modulus (blue), normal force (green), and shrinkage (yellow) as a function of time for the composition containing 0.5 wt% SSQ-SH.

**Figure 9:**
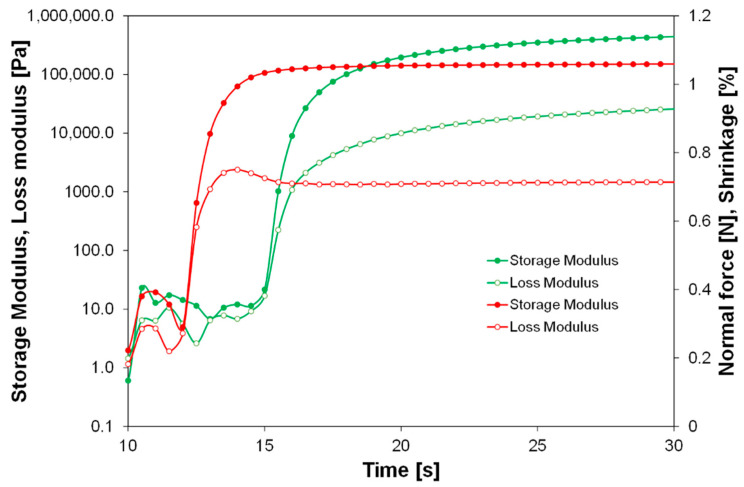
Comparison of the storage modulus and loss modulus as a function of time for a reference composition (green) and a composition containing 50 wt% SSQ-SH (red).

Photoreological studies of the developed compositions were also carried out. The time dependence of the storage modulus, loss modulus, and normal force, as well as the profiles of changes in polymerization shrinkage growth for an example sample containing 0.5 wt% SSQ-SH, is shown in Figure 8. An important parameter in photorheological studies is the gel point, which is the point at which the photopolymerization process starts. Figure 9 presents a comparison of the storage modulus and loss modulus as a function of time for a reference composition (green) and a composition containing 50 wt% SSQ-SH. The gelation time for the composition including 50 wt% SSQ-SH is about 2.5 s shorter than the gelation time for the reference composition. This shows that the addition of SSQ-SH has an effect on accelerating the photopolymerization process. Moreover, the addition of as little as 2 wt% SSQ-SH made the gelation time comparable to that for the composition with 50 wt% SSQ-SH (Table 3). The shrinkage that occurred during the photopolymerization process was also ascertained using the findings of the rheological investigation performed on the systems that were evaluated. Polymerization shrinkage was calculated using Equation (1).

(1) $$Shrinkage\;\left[\%\right]=\left(1-\frac{d_{After}}{d_{Before}}\right)\cdot 100\%$$

where *d_After_* denotes the distance between the glass and aluminum plates after the photopolymerization process [mm], and *d_Before_* is the distance between the glass and aluminum plates before the photopolymerization process [mm].

The determination of polymerization shrinkage is extremely important in both photo-cured 3D printing and dental applications [53]. Polymerization shrinkage is undesirable in both these applications, as marginal gaps can appear, e.g., between filling and tooth tissue, resulting in secondary caries [54]. The shrinkage for composites containing different SSQ-SH contents was determined using Equation (1). The results of this experiment are presented in Figure 8 and Table 3. Under special measurement conditions, the average shrinkage of the samples was less than 1%.

### 3.4. Photo-DSC

**Figure 10:**
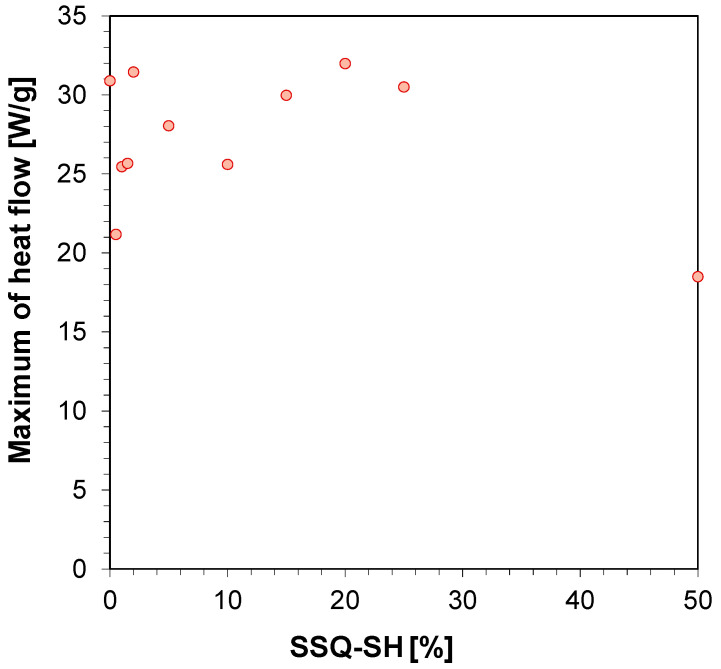
Dependence of maximum amount of heat flow on SSQ-SH content.

**Figure 11:**
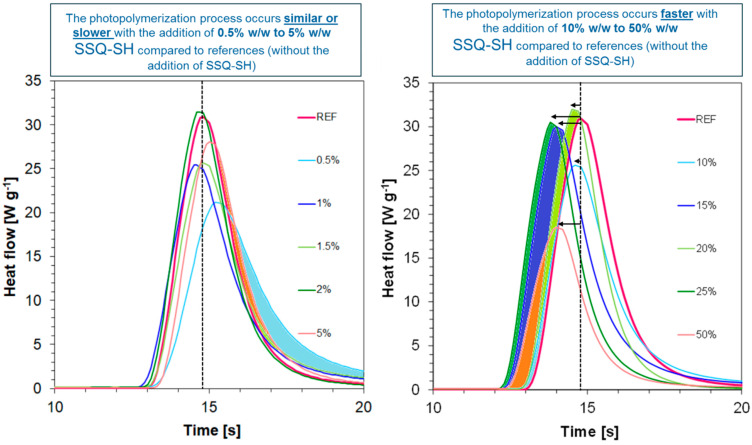
Heat flow time dependence for compositions after photopolymerization.

Similar kinetic studies for the developed compositions were carried out using Photo-the DSC technique. The measurement of the heat amount as a function of time for the studied compositions is presented in Table 3, and the maximum amount of heat is shown in Table 3. Photopolymerization was conducted using the same light source used for the photo-rheological studies. The maximum heat flow for the compositions oscillates in the range from 18.5 W·g^−1^ for a composition containing 50 wt% SSQ-SH to about 30 W·g^−1^ for the other compositions (Table 3, Figure 10 and Figure 11). No significant correlation was observed between maximum heat flow and SSQ-SH content (Figure 10). It is noteworthy, however, that the process for the reference sample itself, for which (3-thiopropyl)polysilsesquioxane was not added, is very fast under the measured conditions, transpiring in less than 20 s. Nevertheless, it was shown that the addition of a small amount of SSQ-SH in the range of 0.5% *w*/*w* to 5% *w*/*w* did not significantly affect the photopolymerization process. The photopolymerization process occurs at a similar speed or even slower with the addition of 0.5% *w*/*w* to 5% *w*/*w* SSQ-SH compared to the same speed for the references (without the addition of SSQ-SH). In contrast, the photopolymerization process occurs faster with the addition of a larger amount of SSQ-SH (10% *w*/*w* to 50% *w*/*w*) compared to the same speed for the references (without the addition of SSQ-SH). This indicates that the compound is involved in the photo-polymerization process.

The addition of SSQ-SH causes an acceleration of gelation and polymerization. In this case, it is the photopolymerization of thiol-ene. The radical photopolymerization of methacrylate or acrylate monomers alone has a number of significant drawbacks. One of the main disadvantages is the inhibition of polymerization by oxygen, as well as high polymerization shrinkage and the formation of highly heterogeneous polymer networks [55,56]. The utilization of the thiol-ene reaction provides an excellent approach to chemical synthesis and the manufacturing of customized materials by combining the advantages of a photoinitiated process with the traditional benefits of click reactions [57]. In contrast to the photopolymerization of (meth)acrylate, the thiol-incorporated procedure proceeds with reduced or zero oxygen inhibition, reduces shrinkage stresses due to the photopolymerization process, and produces an excellent homogeneous polymer network [58].

### 3.5. Water Contact Angle

**Figure 12:**
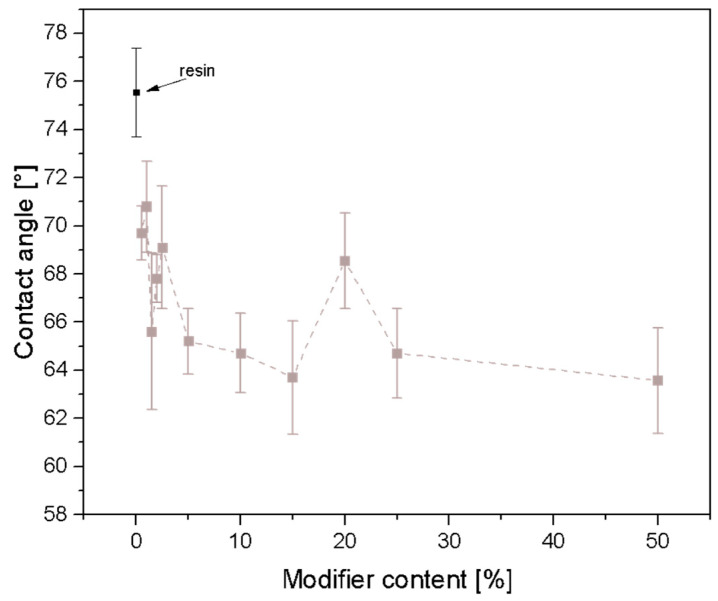
Results of contact angle.

The resin’s water contact angle was determined to be 75.5°. Through testing, it was observed that the hydrophilicity of all the samples increased due to the modification of Ebecryl^®^ resin with SSQ-SH, as depicted in Figure 12. The formation of a new C-S bond and the incorporation of sulfur atoms into the resin matrix can result in a change in polarity, consequently altering the interaction between the coating material and water. As the amount of modifier in the matrix increases, the surface of the composite becomes more receptive to water, leading to a decrease in the contact angle value.

### 3.6. Mechanical Properties

**Figure 13:**
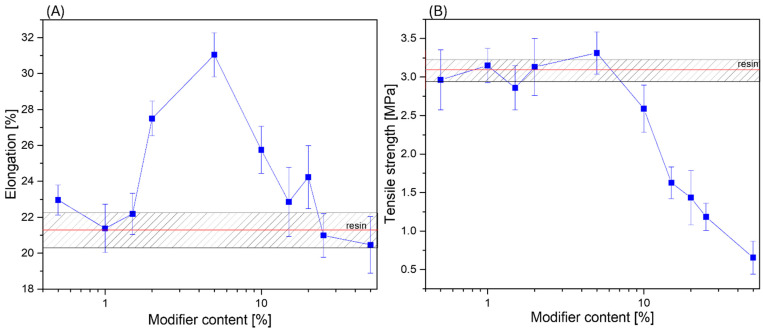
Mechanical properties of composites: (**A**) elongation; (**B**) tensile strength.

Tensile strength and elongation-at-break tests were conducted on standardized samples following ISO standard [43] to precisely evaluate the impact of incorporating (3-thiopropyl)polysilsesquioxane on the mechanical properties of the resin (Figure 13). The reference sample exhibited a tensile strength of 3.1 MPa (red line). The addition of the modifier within the range of 0.5% to 5% did not significantly affect tensile strength, yielding values ranging from 2.85 to 3.31 MPa. However, the elongation at break for samples modified at 0.5–15% concentrations was higher than that for the reference sample (Elongation = 21.2%). The most significant increase was observed in samples containing 2% and 5% modifier, displaying increases of 29.6% and 46.4%, respectively, in comparison to the unmodified resin. Furthermore, a rise in elongation value was noted upon increasing the additive concentration, peaking at 5% for elongation and tensile strength. Beyond these concentrations, further increments in the amount of additive did not increase the analyzed values. In summary, the incorporation of (3-thiopropyl)polysilsesquioxane can effectively enhance the elasticity of resins while preserving their tensile strength, provided an appropriate modifier concentration is selected. The critical concentrations to consider are 5% for elongation and tensile strength. These results underscore the potential of (3-thiopropyl)polysilsesquioxane as a viable tool for improving resin properties. 

## 4. Conclusions

This article presents the preparation of a photocurable urethane-acrylate resin modified with (3-thiopropyl)polysilsesquioxane (SSQ-SH), achieved through a click reaction between the SH groups in the modifier’s structure and the acrylic groups in the resin. The newly developed materials exhibited significantly changed thermal properties, as evidenced by their higher thermal degradation temperatures, onset temperatures, temperatures corresponding to 1% and 5% mass loss, and the temperature at the maximum rate of mass loss (for 50 wt% SSQ-SH, T_5%_ was 310.8 °C, representing an increase of 20.4 °C). Moreover, tests revealed that the incorporation of SSQ-SH accelerated both gelation and polymerization processes, indicating its potential for improving processing efficiency in various applications. The incorporation of (3-thiopropyl)polysilsesquioxane effectively enhances the elasticity of resins while maintaining their tensile strength, particularly at concentrations of 5%. These results underscore the potential of (3-thiopropyl)polysilsesquioxane as a promising tool for enhancing the properties of resins, especially in applications where both thermal stability and mechanical flexibility are important, such as in coatings. Incorporating (3-thiopropyl)polysilsesquioxane into resin formulations could lead to enhanced performance and expanded applications in industries (e.g., automotive, construction) requiring durable and flexible coatings.

## 5. Patents

The results of this publication have been patented with Polish patent application no. P.443618.

## Figures and Tables

**Table 1:** Composite systems.

Modifier Content [%]	SSQ-SH [g]	Resin [g]	Initiator DMPA [g]
0	0	6.00	0.06
0.5	0.03	5.97	0.06
1.0	0.06	5.94	0.06
1.5	0.09	5.91	0.06
2.0	0.12	5.88	0.06
5.0	0.30	5.70	0.06
10.0	0.60	5.40	0.06
15.0	0.90	5.10	0.06
20.0	1.20	4.80	0.06
25.0	1.50	4.50	0.06
50.0	3.00	3.00	0.06

**Table 2:** Results of thermogravimetric analysis.

Modifier Content [%]	Temperature at 1% Mass Change [°C]		Temperature at 5% Mass Change [°C]		Onset Temperature [°C]		Temperature at the Maximum Rate of Mass Loss [°C]		Residual Mass at 600 °C [%]
	N_2_	Δ*T*	N_2_	Δ*T*	N_2_	Δ*T*	N_2_	Δ*T*	N_2_
0	157.8	-	290.4	-	306.0	-	345.3	-	0
0.5	194.0	36.2	300.2	9.8	316.1	10.1	347.9	2.6	1.8
1	167.5	9.7	300.6	10.2	317.1	11.1	349.6	4.3	5.2
1.5	159.8	2.0	301.3	10.9	318.7	12.7	349.2	3.9	6.6
2	161.7	3.9	300.0	9.6	318.8	12.8	352.1	6.8	7.9
5	220.7	62.9	307.7	17.3	319.0	13.0	350.9	5.6	9.5
10	226.4	68.6	308.6	18.2	323.2	17.2	353.5	8.2	10.2
15	162.3	4.5	304.6	14.2	318.0	12.0	352.1	6.8	19.3
20	172.6	14.8	302.4	12.0	319.5	13.5	354.9	9.6	22.6
25	175.3	17.5	301.2	10.8	320.1	14.1	358.7	13.4	23.0
50	233.0	75.2	310.8	20.4	318.7	12.7	367.8	22.5	30.3

**Table 3:** Summary of parameters for the compositions.

Modifier Content [%]	Gel Point [s]	Shrinkage [%]	Heat Flow Max. [W/g]
0	15.0	0.4	30.895
0.5	13.5	0.3	21.175
1	13.0	0.4	25.454
1.5	12.0	0.4	25.675
5	12.5	0.4	32.057
10	12.5	0.3	28.050
15	13.0	0.4	25.602
20	12.5	0.3	31.987
25	12.5	0.3	30.502
50	12.5	0.2	18.499

## Data Availability

Data are contained within the article.
